# Use of Different Natural Products to Control Growth of Titanium Oxide Nanoparticles in Green Solvent Emulsion, Characterization, and Their Photocatalytic Application

**DOI:** 10.1155/2021/6626313

**Published:** 2021-03-12

**Authors:** Eneyew Tilahun Bekele, Bedasa Abdisa Gonfa, Fedlu Kedir Sabir

**Affiliations:** Department of Applied Chemistry, School of Applied Natural Science, Adama Science and Technology University, P.O. Box 1888, Adama, Ethiopia

## Abstract

Water, one of the crucial and the pillar resources to every living thing, could be polluted day to day by different causes such as expansion in industrialization, rapid increment in population size, the threat of climate, and growth of urbanization. The existence of a number of organic dyes, detergents, and pesticides from industrial effluents could lead to severe diseases and even to the death of human beings. Currently, remediation of those hazardous organic contaminants using semiconductor metal oxide catalysts has received extensive attention in recent years. Among the numerous nanometal oxides, titanium oxide (TiO_2_) nanoparticles (NPs) have been well known as a significant photocatalytic material due to their suitable physiochemical behaviors such as stability, conductivity, high surface area to volume ratio, structure, and porosity nature at the nanoscale level. TiO_2_ semiconductor nanoparticles could be synthesized via several physiochemical approaches; among those, the biogenic technique is the most selective one which involves the synthesis of NPs using different templates. Biogenic synthesis of nanoparticles is an environmentally friendly protocol that involves the use of different parts and types of biogenic sources such as bacteria, fungi, yeast, virus, and green plants or the byproducts of their metabolism, which act as both reducing and stabilizing agents. TiO_2_ NPs obtained via the biogenic method provide a potential application for the degradation of organic dyes and other pollutants in wastewater. This method of synthesis of NPs has been given a great attention by researchers due to their nontoxicity, low cost, environmental friendliness, the usage of green solvents, and simplicity of the process. This review focuses on summarizing the synthesis of TiO_2_ NPs using various biogenic sources, characterization, and their photocatalytic applications for the degradation of different wastes and organic dyes from polluted water.

## 1. Introduction

In the past decade, there has been a marked increase in the field of synthesis of various nanoparticles such as metallic, metal oxide, nanocomposite, and decorated nanoparticles with controlled morphologies and remarkable features, making them an extensive area of research. The possibility of synthesizing NPs with controlled particle size, shape, and crystalline nature enables NPs to be used for various potential applications, such as degradation of organic pollutants in wastewater, biomedical, biosensor, catalyst for bacterial biotoxin elimination, and for the photoanode in dye-sensitized solar cells (DSSCs) [1–3]. Among the various adapted techniques for the synthesis of nanoparticles, biogenic synthesis of nanoparticles is an environmentally friendly protocol that involves the use of different parts and types of biogenic natural sources such as bacteria (intracellular and extracellular), fungi, yeast, virus, and different parts of green plants (flower, stem, root, leaf, and fruit) or the byproducts of their metabolism, which act as both reducing and stabilizing agents to prevent the overall growth of nanoparticles during their synthesis process. Moreover, metal oxide nanoparticles such as TiO_2_ NPs obtained via this biogenic synthesis method, a greener approach synthesis method, are safe, nontoxic, ecofriendly, and achieved with minimum energy [2, 3].

The natural environment suffers serious hazardous problems due to the release of different waste materials to the natural environment such as the accumulation of agricultural fruit and vegetable waste and industrial wastes (organic dyes, pesticides, inorganic contaminants and detergents) and due to the decomposition of toxic and poisonous gases and chemical species produced from chemical industries. Their toxicity and stability to natural decomposition and persistence in the environment have been the cause of much concern to societies and regulation authorities' worldwide. With the rapid development of industrial society and increments in population size, the threat of climate change and increasing environmental-safety threats pose a major concern. Nowadays, water pollution is becoming one of the most serious problems in the environmental fields. Wastewater effluents released from different industries such as textile, rubber, paper, and plastic contain several kinds of synthetic dyestuffs that could result in severe diseases in humans, animals, and plant species too [4, 5].

Previously, a number of different physiochemical techniques were adapted for the treatment of wastewater such as sedimentation-flocculation, coagulation, molecular sieving, ion exchange, reverse osmosis, membrane filtration, ozonation, chlorination, chemical precipitation, adsorption, photocatalysis, chemical methods, and electrochemical techniques, and many others were used to remove toxic organic/inorganic pollutants from polluted water [4–7]. However, a majority of those techniques are not efficient enough and adequate in terms of cost, energy, environmental friendless, and purification capability, and they need expensive and complicated methods and have slow adsorption rate, as well lack complete degradation of contaminants [6]. In addition to this, most organic dyes, detergents, and pesticide compounds ordinarily contain benzene and naphthalene rings that cannot be decomposed easily by conventional physical, biological, and chemical methods. Among these protocols from advanced oxidation processes, heterogeneous photocatalysis assisted by semiconductor nanometal oxides such as TiO_2_ has been suggested as a design which is simple, operates under ambient conditions, has high stability, and cost effective, energy efficient, and environmentally safe. Also, the methods do not result in the formation of secondary pollution to the natural environment [5–7].

**Figure 1:**
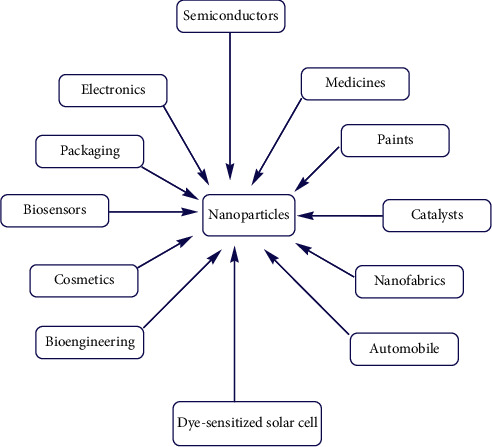
An overview of applications of nanoparticles [9].

This is due to the fact that metal oxide semiconductor nanomaterials are inexpensive, nontoxic, possess high surface area to volume ratio, exhibiting tunable properties which can be modified by size reduction, doping, sensitizers, and composite form, affording facility for the multielectron transfer process, and capable of extended use without substantial loss of photocatalytic activity [8]. Different metal oxide materials at the nanolevel possess unique biological, optical, magnetic, mechanical, thermal, catalytic, and electrical properties and also due to the fact that as the size of the nanoparticles decreases, the surface area to volume ratio increases, which is suitable for the process of photocatalysis [6–9]. In general, various semiconductor oxide nanoparticles play an important role in various applications (Figure 1) such as self-cleaning, gas sensors, optics, photoelectrochemical devices, solar cell applications, photocatalysis, cosmetic industry, antibacterial activity, antifungal activity, antioxidant activity, and medicinal applications [10].

**Figure 2:**
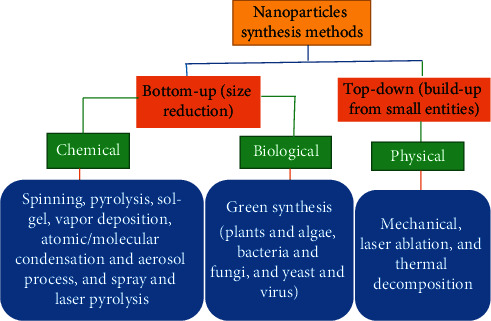
Synthesis methods of nanoparticles [3].

Among the various metal oxides, titanium dioxide (TiO_2_) NPs are an inert, nontoxic, and inexpensive material, whose high refractive index and high capability to absorb UV light make it an interesting white pigment and environmentally friendly catalyst. The nanosized TiO_2_ NPs are widely used to provide whiteness and opacity to products such as sunscreen lotions, paints, plastics, papers, solar cell, medicinal applications, inks, food colorants, and toothpastes. There are various methods to synthesize different metal oxide nanoparticles having various applications. According to research reports, titanium dioxide NPs could be synthesized through chemical vapour deposition [11], microemulsion [12], chemical precipitation [13], and hydrothermal [14, 15], solvothermal [16], sol-gel [17], electrochemical [18], and green [19] methods. Among the mentioned methods, green synthesis of nanomaterials is getting increased attention because of its simplicity, fastness, ecofriendliness, and nontoxic nature, and it involves the use of green solvents such as distilled water and ethanol and an economical approach [1–3]. It involves three important steps during the synthesis process such as solvent medium selection, environmental benign reducing and capping agent selection, and nontoxic substances for nanoparticles stability selection [20–22].  Figure 2 summarizes the various synthesis techniques for different nanoparticles.

Earlier studies show that the use of toxic and expensive chemicals as reducing and capping agents resulted in the production of larger particles, which again consumes extra energy and provides large particle size. Also, the chemically synthesized nanoparticles were described to exhibit less stability and added agglomeration and aggregation [23–28]. As a result, there is a necessity to improve and develop an ecofriendly protocol synthesis method that will create dispersible and stable nanoparticles of manageable size, which consumes less energy, uses green solvents, and involves easily controllable techniques. In the light of the aforementioned issues and facts in mind, this review work summarizes the recent advancement in the biogenic synthesis of TiO_2_ NPs using various medicinal plant part extracts and microorganisms such as bacteria, fungi, algae, and yeast, characterization, and their photocatalytic applications.

So many research works were reported concerning synthesis and photocatalytic activity of titanium oxide nanoparticles. Up to the knowledge of the authors, no work was reported that summarizes on the synthesis of TiO_2_ NPs using different sources of natural products such as different parts of green plants, bacteria (both Gram positive and Gram negative), different species of fungi (synthesized either intracellularly or extracellularly), and yeasts for the treatment of wastewater. The novelty of this work also lies on showing the possibility of production of TiO_2_ NPs with different shapes and sizes following the change in the type and nature of the stabilizing/reducing role of plant extract metabolites during synthesis [29–33]. The metabolites used during nanoparticle synthesis prevent agglomeration and control the sizes of nanoparticles. It is well reported that the size of TiO_2_ NPs could significantly affect the photocatalytic degradation of organic dyes [34]. High-surface-area (small-sized) NPs of titanium oxides are highly recommended for photocatalytic degradation of dye pollutants in waste water [32–35].

## 2. Biogenic Synthesis Methods of Titanium Oxide Nanoparticles

Green chemistry and biological processes have led to the development of an environment-friendly process for the synthesis of TiO_2_ nanoparticles. A vast array of biological resources available in nature including living plants, plant products and plant extracts, algae, fungi, yeast, bacteria, and viruses could all be employed for the synthesis of TiO_2_ nanomaterial. Biological methods are considered and regarded as safe, cost-effective, biocompatible, nontoxic, sustainable, and environment-friendly processes. In addition, most bioprocesses occur under normal air, pressure, and temperature, resulting in vast energy savings, high yield, and low cost [36].

### 2.1. Algae-Mediated Biogenic Synthesis of Titanium Oxide Nanoparticles

**Figure 3:**
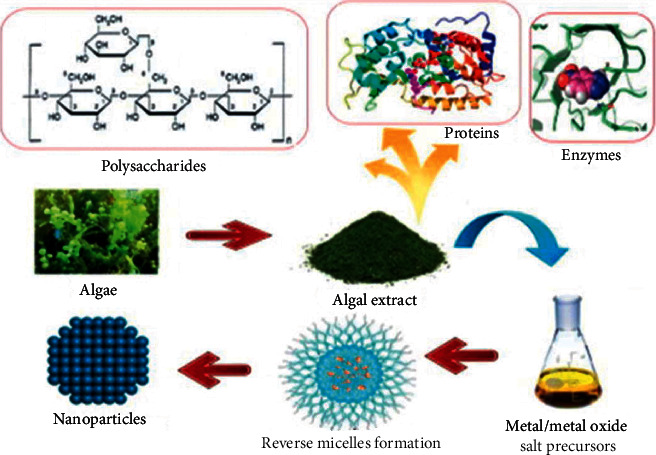
Algae-mediated synthesis of metal/metal oxide NPs [37].

Algae are aquatic phototrophic, and recent studies have shown that some of them are not only accumulate heavy metals but are also used to biologically synthesize metallic nanoparticles. The use of algae for biosynthesis of TiO_2_ nanoparticles has become prevalent during these days due to their easy access and efficacy. The biomolecules such as enzymes, proteins, polysaccharides, and others present in the algal extract have not relatively been well exploited for nanoparticle synthesis compared to similar other natural sources such as plants and bacteria [37].  Figure 3 shows the scheme for algae-mediated synthesis of metal/metal oxide NPs including TiO_2_ NPs.

### 2.2. Fungi-Mediated Biogenic Synthesis of TiO_2_ Nanoparticles

The first report of using fungi extract to biosynthesize nanoparticles dates back to a letter in nature since 1989. Fungi have attracted more attention regarding the research on biological production of TiO_2_ nanoparticles due to their toleration and metal bioaccumulation capability, as well as their ability to secrete large amounts of enzymes [38, 39]. Previously, TiO_2_ NPs were synthesized from a TiO_2_ precursor by employing *Aspergillus tubingensis* fungi [40]. The study reported that the dynamic light scattering (DLS) calculated size was estimated between 1.5 and 30 nm with a polydispersity index value of 0.194. Similarly, TiO_2_ NPs were synthesized in the presence of bulk TiO_2_ as a precursor using *Aspergillus niger* fungi extract. They reported that the obtained TiO_2_ NPs' size was in the range of 73.58 to 106.9 nm as it was determined via SEM [41]. It was reported that fungi are ideal biocatalysts for TiO_2_ NPs biosynthesis, in contrast to bacteria, as they are well known for producing greater amounts of biologically active substances that make the fungus more appropriate for large-scale production of different nanomaterials too [37].

### 2.3. Bacteria-Mediated Biogenic Synthesis of TiO_2_ Nanoparticles

In contrast to other kinds of microorganisms, bacteria were utilized to synthesize nanoparticles earlier. Due to the mild conditions, high yield, and easy purification and manipulation, bacteria become the most widely studied microorganism, with the title of “the factory of nanomaterials” [42]. Previously, different researchers made use of bacteria as a reducing and a capping agent to control the grain/overgrowth during the synthesis of titanium dioxide nanoparticles and other nanomaterials having various applications [42]. TiO_2_ NPs were synthesized in the presence of *Aeromonas hydrophila* bacteria using the bulk form of TiO_2_ as a precursor. The XRD pattern of the synthesized TiO_2_ NPs estimated the size as 40.50 nm having spherical morphology as it was confirmed from FESEM analysis [43]. Moreover, a research report show that TiO_2_ NPs were synthesized in the presence of *Planomicrobium spp.* bacteria using the bulk form of TiO_2_ as a precursor [44, 45]. The XRD crystal size of the obtained TiO2 NPs measured was 8.89 nm. In addition, TiO_2_ NPs were synthesized using the bacteria *Bacillus mycoides* in the presence of titanyl hydroxide as the precursor. The obtained TiO_2_ NPs produced were predominately anatase crystal with a polydisperse size of 40–60 nm [46]. Advantages of using a bacterial system include easy handling and hereditary manipulation without much difficulty. As it was stated by different scholars, bacteria have been employed in commercial biotechnological processes such as bioleaching and bioremediation. Bacteria possess remarkable abilities to reduce heavy metal ions and are potential candidates for TiO_2_ nanoparticles synthesis [42, 47].

### 2.4. Plant-Extract-Mediated Biogenic Synthesis of TiO_2_ Nanoparticles

**Figure 4:**
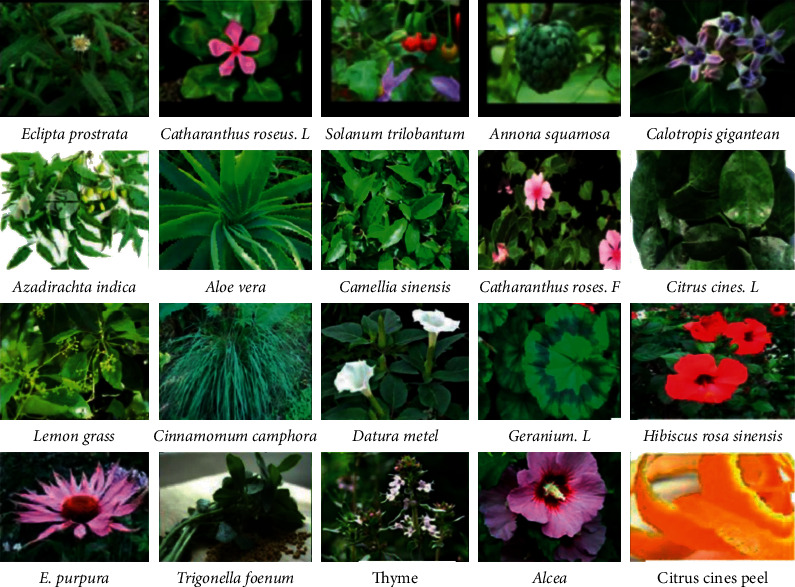
Some selected types of plants used as a template for TiO_2_ NPs synthesis [36, 37].

Even though different microorganisms have been employed as an alternative template for the synthesis of nanomaterials instead of using expensive and toxic organic solvents and chemicals as a stabilizer, the use of microorganisms has its own disadvantages as compared to medicinal green plants as they require advanced equipments and instruments to culture and enhance their growth before they are used for synthesis purpose and require optimization of different parameters to achieve and maintain their growth and to be used during the synthesis process. Instead, incorporating different parts of green and medicinal plants results in too ecofriendly benefits to the ecosystem. Due to the diversity of plants, nanoparticles synthesis from plant extract has known as an interesting subject across the world as different plant species are being rapidly investigated and used in nanoparticles synthesis. The use of plant extracts to synthesize TiO_2_ nanoparticles is receiving attention in recent times because of simplicity, eco-friendly approaches, cost effectiveness, readily-scalable processes, and much product formed with the minimum cost [36].  Figure 4 shows some of the selected plant types and the different parts that were used as a template during TiO_2_ NPs synthesis.

TiO_2_ NPs were synthesized by the reaction between titanium tetraisoproxide and ethanolic leaf extract of *Nyctanthes arbor-tristis*. XRD result analysis revealed that the average grain size was near 100 nm having spherical morphology [48]. In our previous work [49], we have reported on the synthesis of TiO_2_ NPs using titanium tetrabutoxide as a precursor in the presence of ethanolic root extract of *Kniphofia foliosa* as a template. The obtained XRD analysis shows the average crystalline size was estimated between 8.2 and 10.2 nm for the different volume ratios, and the FESEM result shows the obtained nanoparticles have spherical morphology. Similarly, using the TiO (OH)_2_ precursor and aqueous extracts of *Eclipta prostrata* leaves as a biotemplating agent, NPs were synthesized. The obtained TiO_2_ NPs were spherical in shape, and their size ranged from 36 nm to 68 nm [50].

**Figure 5:**
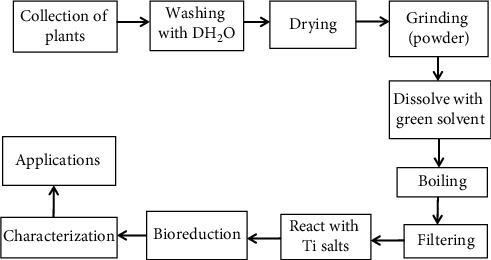
TiO_2_ NPs synthetic pathway using plant extracts as green sources [53].

Previously, TiO2 NPs were synthesized using the aqueous extract of *Psidium guajava* leaf and TiO (OH)_2_ as a precursor. They reported that the average size of plant-mediated synthesized TiO_2_ NPs was estimated as 32.58 nm [51]. Using the titanium (iv) isopropoxide precursor, TiO_2_ NPs were also synthesized in the presence of ethanolic extract of *cassia auriculata* leaves [52]. FESEM revealed that the average particle size obtained was from 38 to 44.2 nm. In a similar fashion, TiO_2_ NPs were synthesized using aqueous extract of orange peel as a reducing agent and titanium (iv) isopropoxide solution as a starting material, and the average crystallite size of the obtained TiO_2_ NPs was calculated as 19 nm [53]. It has been reported that TiO_2_ NPs were synthesized using aqueous extract of *C. giganta* flower and TiO (OH)_2_ was used as a precursor. SEM micrograph analysis of the obtained TiO_2_ NPs showed aggregation and spherical shape with an average size between 160 and 220 nm [29]. TiO_2_ NPs were prepared from the aqueous leaf extract of *Ocimum basilicum L. var. Benth* using titanium (iv) isopropoxide as a precursor. The particle size of the synthesized TiO2 NPs was found as 6.97 nm as per Debye Scherrer [54]. In general synthesis of TiO_2_ NPs using different parts of medicinal and nonmedicinal green plants, the path depicted in Figure 5 is followed.

It has been reported that phytochemicals and primary and secondary metabolites are well known as natural resources that are responsible for metal salt reductions during the green synthesis of TiO2 NPs [55]. The green-synthesized TiO_2_ NPs show the best photocatalyst activity due to their long thermodynamic stability and strong oxidizing power.  Table 1 summarizes the biogenic synthesis of TiO_2_ NPs in the presence of different types of templates.

The table shows reports on biogenic synthesized TiO_2_ NPs from different templates as a biological source. Due to the biodiversity of plants, bacteria, yeast, and fungi, TiO_2_ NPs with different particle sizes, morphologies, and applications can easily be synthesized in large amount even at an industrial level [55].

## 3. Characterization Techniques Used for Bioassisted Synthesized TiO_2_ NPs

In order to check and confirm the formation of the synthesized TiO_2_ NPs for the desired applications, a number of physiochemical and surface analysis techniques were carried out, such as morphological characterizations (scanning electron microscopy (SEM) and transmission electron microscopy (TEM)) and structural characterizations (X-ray diffraction (XRD), energy-dispersive X-ray (EDS), X-ray photoelectron spectroscopy (XPS) used for surface composition, chemical states, elemental ratio, and exact bonding nature analysis, Fourier-transform infrared (FT-IR) which provides different information about functional groups that play great role during the synthesis process, Raman spectroscopy, and Brunauer–Emmett–Teller (BET) used for the determination of surface area, pore diameter, pore size, and pore volume of the formed TiO_2_ NPs). Total reflection X-ray fluorescence analysis (TXRF) can also be adapted to deal with elemental composition of the biogenic synthesized TiO_2_ NPs. Particle size and surface area determination can be carried out using SEM, TEM, DLS, and XRD techniques. Optical and light-absorbing behaviors can be carried out using UV-Vis, PL, and DLS techniques. In addition to these techniques, electrochemical characterization can be performed using CV to determine the electronic band-gap energy. Thermal stability and behavior of the as-synthesized TiO_2_ NPs could be characterized using TGA/DTA or TGA/DTG before the calcination step was performed.

### 3.1. Characterization by UV-Vis Absorbance Spectroscopy

**Figure 6:**
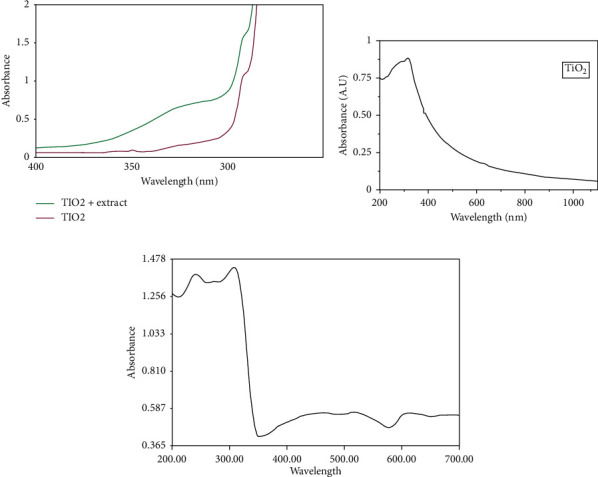
UV-Vis absorption spectra of (a) *Echinacea purpurea herba*-, (b) *Hibiscus* flower-, and (c) *Aloe vera*-extract-mediated synthesized TiO_2_ NPs [68, 69, 73].

UV-Vis absorption spectroscopy is an important technique to monitor the formation and stability of titanium dioxide nanoparticles in aqueous solutions and to characterize optical properties of TiO_2_ NPs. The absorption spectrum of TiO_2_ NPs is sensitive to several factors, including particle size, shape, and particle-particle interaction (agglomeration) with the medium. The absorption maximum (*λ*_max_) of the synthesized nanoparticles depends on its size and shape [83, 84]. Figures 6(a)–6(c) show the absorption spectra of *Echinacea purpurea herba*-, *Hibiscus* flower-, and *Aloe vera*-extract-mediated synthesized TiO_2_ NPs, respectively.

Figure 6(a) shows the UV-Vis absorption spectra for *E. purpurea herba*-mediated TiO_2_ nanoparticles between 200 and 400 nm. The absorption of TiO_2_ appears at 280 nm in UV-Vis spectroscopy. Similar studies were also reported by Roopan et al. [85].  Figure 6(b) shows the absorbance of synthesized TiO_2_ NPs using *Hibiscus* flower extract as a template. The spectra exhibit a range between 200 and 1200 nm and the peak at 354 nm wavelength with an absorbance of 0.86 (<1), which means that it exhibits good absorbance at the UV region, as it was reported by Vijayalakshmi and Rajendran [86], and the energy band gap was estimated at 3.503 eV.  Figure 6(c) proves the UV-Vis absorbance spectra of *Aloe vera* extract biogenic-synthesized TiO_2_ NPs. The absorption spectra of TiO_2_ nanoparticles exhibit strong absorption below 400 nm. The spectrum of the TiO_2_ sample calcined at 400°C indicates the absorption onset at around 393 nm with a band gap energy of 3.196 eV, which is in excellent agreement with the band gap of the anatase phase [87].

### 3.2. Characterization Using XRD

**Figure 7:**
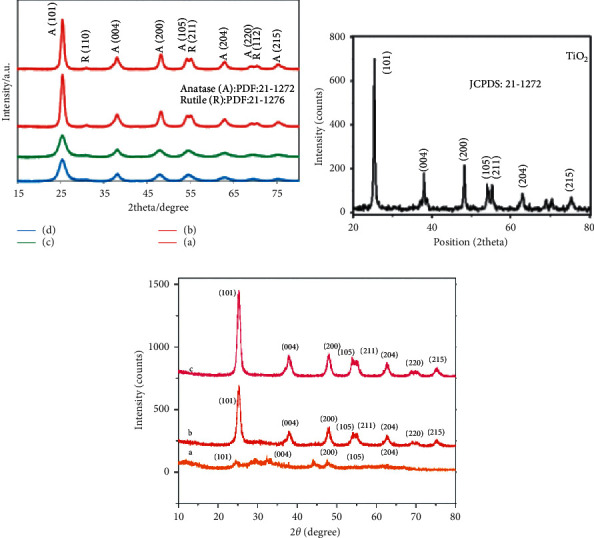
(a) Thyme (a), *Alcea* (b), and *Alcea* calcined at 500°C (c), and *thyme* calcined at 500°C (d), (b) *Moringa oleifera*-leaf-, and (c) root of *Kniphofia foliosa-* (a, 1 : 2, b, 1 : 1, and c, 2 : 1) extract-mediated synthesized TiO_2_ NPs [49, 67, 71].

The X-ray diffraction measurement analysis technique could provide information about the structure, phase, and average crystalline size of the formed nanoparticles. Figures 7(a)–7(c) depict *Alcea*-, thyme-*, Moringa oleifera*-leaf-, and *Kniphofia foliosa*-root-extract-mediated synthesized TiO_2_ NPs, respectively. As shown in Figure 7(a), the XRD patterns of *Alcea-* and thyme-extract-assisted synthesized TiO_2_ NPs indicate the sharp peaks at a 2*θ* value of ≈25.54, 37.39, 47.84, 53.69, 62.12, 68.28, and 74.55, which belong to (101), (004), (200), (105), (204), (220), and (215) planes of the anatase phase as supported by the Joint Committee on Powder Diffraction Standards (JCPDS) card number of 21–1272, respectively. In addition to this, the observed peaks at a 2*θ* value of 30.48, 54.58, and 69.89 are related to the diffraction of (110), (211), and (112) plates of the rutile phase, respectively, according to their JCPDS card number of 21–1276, as supported by the work of Hajalilou et al. [88]. From the XRD analysis result, the average crystallite size for thyme*-* and *Alcea*-mediated synthesized TiO_2_ NPs was found to be about 6 and 10 nm, respectively.

Figure 7(b) proves *Moringa oleifera*-leaf-extract-based synthesized TiO_2_ NPs, and in the XRD analysis, five diffraction peaks were observed ≈2*θ* at 25.3, 37.7, 48.1, 54.0, and 62.7 with their corresponding miller indexes values of (101), (004), (200), (105), and (204), respectively, having tetragonal body-centered titanium dioxide as confirmed by the JCPDS card number of 86–1156 [89]. The average crystalline size was estimated at 12 nm, which was calculated by using Scherrer's formula. Again, Figure 7(c) displays the X-ray diffraction pattern of root of *Kniphofia foliosa* template synthesized TiO_2_ NPs within different volume ratios of the extract and precursor salt. The diffraction peaks were observed at 2*θ* values of ≈25.3, 38.0, 47.9, 53.2, 54.8862, 62.7, 70.2, and 75.0 along with their miller index planes of (101), (004), (200), (105), (211), (204), (220), and (215), respectively.

The analysis confirms that the biosynthesized nanoparticles are in tetragonal crystal structure without any impurities within the detection limits of XRD as reported in [49]. The average crystalline size for the different kinds of TiO_2_ NPs was estimated at 10.2, 8.2, and 8.5 nm for the 1 : 2, 1 : 1, and 2 : 1 volume ratio of the extract and precursor salt, respectively. As it can be observed from the XRD spectrum, TiO_2_ NPs are synthesized within the volume ratio of 1 : 2, and the crystalline nature is lost due to the addition of an excessive amount of *Kniphofia foliosa* extract beyond the coating surface of TiO_2_ NPs available.

### 3.3. Characterization Using SEM

**Figure 8:**
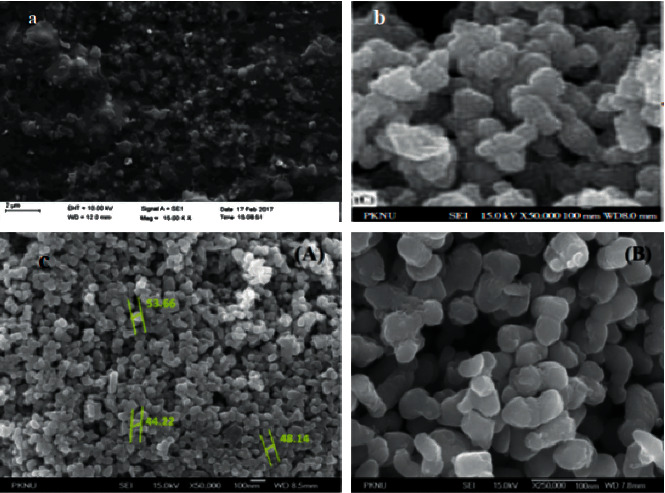
SEM image of (a) *citrus lemon* leaf, (b) *psidium guajava*, and (c) *aeromonas hydrophila* (A, 50000× and B, 2500×) templated synthesis of TiO_2_ NPs [43, 51, 70].

Another very important characterization technique that could provide surface morphology and topography of the biotemplated synthesized TiO_2_ NPs is field-emission scanning electron microscopy (FESEM). This analysis technique allows to determine the peculiar application of green-synthesized TiO_2_ NPs. Figures 8(a)–8(c) (containing (A) and (B) with different magnifications) display the SEM image of citrus lemon-leaf-, *Psidium guajava-*, and *Aeromonas hydrophila*-assisted synthesized TiO_2_ NPs, respectively.

As it can be seen in Figure 8(a), the surface morphology of citrus lemon-leaf-supported biogenic synthesized TiO_2_ nanoparticles was measured by FE-SEM. The micrograph shows nanoscaled TiO_2_ NPs with detailed surface morphology. The results of SEM revealed the development of titanium nanoparticles with different shapes at 15000 X magnification [70]. Figure 8(b) displays the FESEM images of *Psidium guajava-*mediated synthesized TiO_2_ NPs, and the topographical analysis was performed based upon the surface study. Synthesized TiO_2_ NPs were smooth and spherical in shape. The images showed the synthesized nanoparticles in 50000× resolution, which clearly gives physical morphology, particle size, and aspect ratio [51].

Again, Figure 8(c) proves the *Aeromonas hydrophila*-bacteria-mediated synthesized TiO_2_ NPs. Also, the surface morphology of nanoparticles was investigated using the FESEM analysis technique. The nanoparticles were found to be distributed uniformly on the surface with the formation of aggregated nanoparticles. It shows that the particles were densely dispersed with a narrow range of dispersion range [43]. Particles were nanosized with smooth surface morphology. In Figure 8(c), (A) and (B) show that the size of the synthesized TiO_2_ NPs was found to be very consistent.

### 3.4. Characterization Using TEM

**Figure 9:**
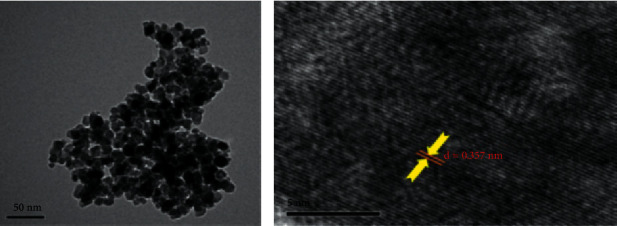
TEM micrograph of (a) *Trigonella foenum graecum-* and (b) *Aloe vera*-mediated synthesized TiO_2_ NPs [68, 74].

The morphology and structural arrangement of biotemplated assisted synthesized TiO_2_ NPs could be perceived through TEM analysis. This analysis could carry out to ascertain and gain further information about the nature of the formed nanoparticles. Figures 9(a) and 9(b) display TEM micrographs of *Trigonella foenum graecum- and Aloe vera*-leaf-extract-mediated synthesized TiO_2_ NPs.

In Figure 9(a), HR-TEM images display spherical shaped polydisperse nanoparticles, and the average particle size of *Trigonella foenum graecum*-assisted TiO_2_ NPs was 20 nm. The TEM micrograph clearly exemplifies the individual nanoparticles formed were nearly in a spherical form with a dimension of 40–60 nm [90]. This investigational analysis indicates it is possible to get small-sized and efficient anatase TiO_2_ nanoparticles via the biogenic method for the desired applications. Similarly, Figure 9(b) shows that TiO_2_ nanoparticles synthesized through the biogenic method using *Aloe vera* crude extract were found to have a crystalline nature. Also, the calculated d-spacing value was estimated as *d* = 0.357 nm, and the value obtained is nearly equal to the XRD d-spacing value [74].

### 3.5. Characterization Using FT- IR Spectroscopy

**Figure 10:**
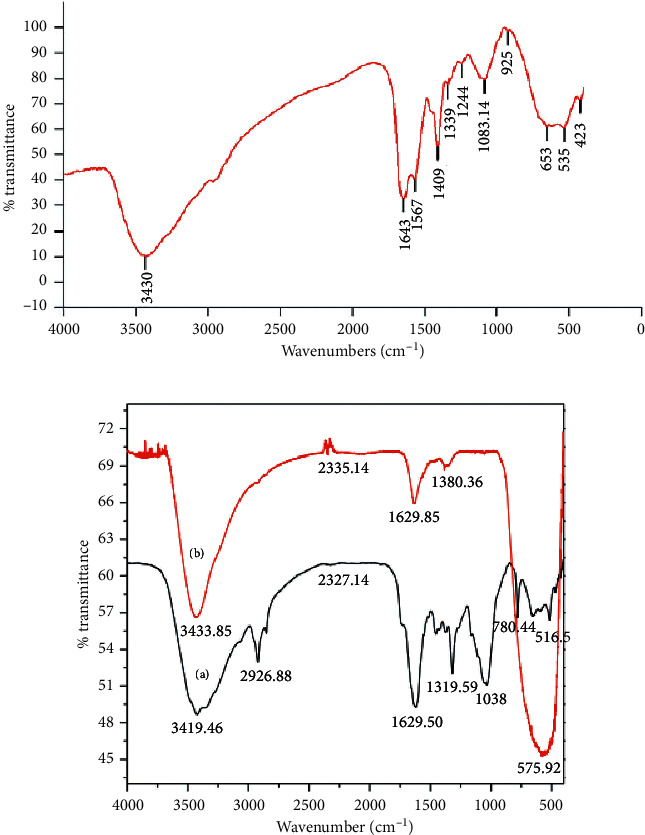
FT-IR spectra of (a) *Aeromonas hydrophila-* and (b) *K. foliosa*-assisted (a, FT-IR of extract and b, extract + TiO_2_) synthesized TiO_2_ NPs [43, 49].

The Fourier-transform infrared spectroscopy analysis technique could provide the necessary information about bioactive ingredients/molecules from the natural product that play a great role as a template during the synthesis process to prevent the overgrowth, aggregation, and then, to maintain phase stability of TiO_2_ NPs. Figures 10(a) and 10(b) show the FT-IR spectra of *Aeromonas hydrophila- and Kniphofia foliosa*-mediated biogenic synthesized TiO_2_ NPs, respectively.

The FT-IR spectra of *Aeromonas hydrophila*-supported synthesized TiO_2_ NPs are indicated in Figure 9(a). The FT-IR spectrum showed characteristic bands at 3430 and 1643 cm^−1^ which correspond to the surface water and hydroxyl group. The band intensities of the spectrum for the synthesized TiO_2_ NPs are 3430, 2937, 1643, 1403, and 1079 cm^−1^. These results indicated that alcohols, phenols, primary amines, lactones, and aliphatic amines are present in *A. hydrophila* which may have been participated in the synthesis process and, as a result, can maintain phase stability. As supported by the report of Coenen et al. [91], functional groups associated with these were the cause for bioreduction of the precursor salt into TiO_2_ NPs.

Figure 10(b) consists of the FT-IR spectra of both the root extract of *k. foliosa* (a) and extract with TiO_2_ (b) NPs. Absorption bands at 3419.46, 2926.88, 1635.14, 1319.59, 1038, and 780.44 cm^−1^ are due to O–H bond stretching, C–H bond stretching of alkanes, C=O bond stretching of carbonyl groups/C=C bond stretching at *α-*, *β*-unsaturated ketone, C–C bond stretching at the aromatic ring, C–O bond bending vibration on phenolic compounds, C–O bond stretching of the hydroxyl group, and out-of-plane C–H bending at the aromatic ring, respectively, indicating the presence of organic compounds such as knipholone anthrone, anthraquinone, and chrysophanol which were used as a capping and reducing agent [92]. The broad absorption band observed at 3433.85 cm^−1^ represents O–H bond stretching due to adsorbed moisture on the surface of the synthesized TiO_2_ NPs. The weak absorption band located at 2335.14 cm^−1^ is due to C=O bond stretching that could be emanated from the presence of adsorbed carbon dioxide on the surface of the NPs. The absorption band at 1629.58 cm^−1^ could be due to carbon dioxide and/or due to O-H bending of molecularly adsorbed water [91]. The broad band centered at 567.13 cm^−1^ represents a characteristic peak of the Ti-O-Ti bending mode of vibration, indicating formation of metal oxygen bonding [93, 94]. The absence of bands at 2926.88, 1635.14, 1319.59, 1038, and 780.44 cm^−1^ in Figure 10 (b, extract + TiO_2_ NPs) shows that organic molecules have been removed from TiO_2_ NPs upon the calcination process.

## 4. Photocatalytic Applications of Biogenic Synthesized TiO_2_ NPs

Photocatalysis is a reaction which uses light to activate a substance which modifies the rate of a chemical reaction without itself being involved. Photocatalytic oxidation (PCO), also known as heterogeneous photocatalysis, has been used since the mid-1970s to decontaminate water from harmful microorganisms, dyes, detergents, pesticides, and inorganic pollutants too [95]. The most commonly used and researched semiconductor nanophotocatalyst TiO_2_ is used as the substrate, and H_2_O and O_2_ are used as an adsorbate. The primary criteria for an efficient semiconductor photocatalyst are that the redox potential of the charge couple, i.e., e/h^+^, lies within the band gap domain of the photocatalyst. A molecular description of a general photocatalytic reaction process involving TiO_2_ is given by the following equations [96]:

(1) $$\begin{array}{c} {\text{TiO}}_{2}+hv⟶{\text{TiO}}_{2}\left({\text{e}}_{cb}^{-}+h_{vb}^{+}\right), \end{array}$$

(2) $$\begin{array}{c} {\text{TiO}}_{2}\left({\text{e}}_{cb}^{-}+h_{vb}^{+}\right.⟶{\text{TiO}}_{2}+\text{heat,} \end{array}$$

(3) $$\begin{array}{c} {\text{TiO}}_{2}+{\text{H}}_{2}{\text{O}}_{\text{ads}}⟶{\text{TiO}}_{2}+{\text{OH}}^{-}+{\text{H}}^{+}, \end{array}$$

(4) $$\begin{array}{c} {\text{e}}_{cb}^{-}+O_{2\text{ads}}⟶O_{2}^{-}, \end{array}$$

(5) $$\begin{array}{c} h_{vb}^{+}+{\text{OH}}^{-}⟶{\text{OH}}^{-}, \end{array}$$

(6) $$\begin{array}{c} \text{OH}+\text{Organic pollutants}⟶{\text{CO}}_{2}+{\text{H}}_{2}\text{O}+\text{others,} \end{array}$$

where CB is the conduction band, VB is the valence band, and hv is the photon energy. Figure 10 shows the schematic representation of basic working principles of photocatalysis.

**Figure 11:**
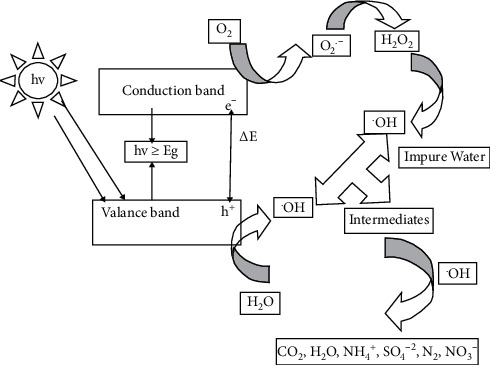
Basic principles of photocatalysis [95].

As it is tried to address in Figure 11, photons with energies greater than the band-gap energy (*E*_g_) can result in the excitation of valence band (VB) electrons which then promote the possible reactions with organic pollutants. The absorption of photons with energy lower than *E*_g_ or longer wavelengths usually causes energy dissipation in the form of heat. The positive hole in the valence band oxidizes either pollutant directly or water to produce hydroxyl radical ^∙^OH, whereas the electron in the conduction band reduces the oxygen adsorbed on the photocatalyst (TiO_2_). The performance of TiO_2_ as a photocatalyst is closely related to its crystal phase, particle size, pore structure, and other morphological properties. Therefore, photocatalytic degradation (PCD) of contaminants using TiO_2_ as a photocatalyst have been under study for disinfection, air purification, environmental cleaning, and wastewater treatment in daily life and industrial activities [95].

The photocatalytic degradation technique with TiO_2_ NPs is generally applied for treating wastewater containing organic and inorganic contaminants due to its ability to achieve complete mineralization of the organic contaminants under mild conditions such as ambient temperature and ambient pressure [97, 98]. In particular, dye degradation is almost essential for wastewater treatment due to its sever toxicity effect. Among the three polymorphs of TiO_2_ NPs (Anatase, Rutile, and Brookite), green-synthesized anatase TiO_2_ exhibits high photocatalytic activity due to high absorption capacity towards organic, molecular oxygen and low rate of recombination of electron hole pairs (EHP), and due to its antifogging effect, it removes bacteria and different harmful organic materials from water [99]. Reporters state that wastewater treatment by TiO_2_ photocatalysis has some benefits compared to conventional water treatment methods. The most important is that it can completely mineralize almost any chemicals and biological compounds, instead of just transferring them to another state [95].

**Figure 12:**
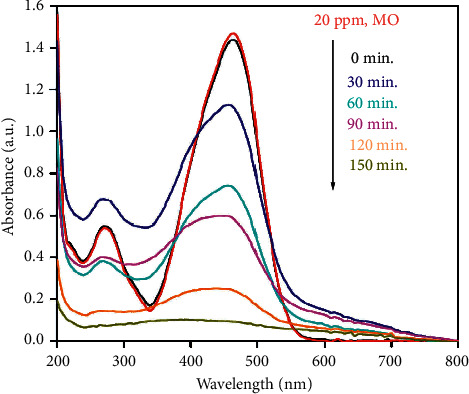
Degradation of MO using PVP-biogenic-assisted synthesized TiO_2_ NPs [101].

Recently, the application of TiO_2_ as a photocatalyst for the removal of organic, inorganic, and other pollutants has attracted many researchers' attention. Previous researchers reported that chlorinated pesticides could be degraded and mineralized through the photocatalytic reaction of TiO_2_ under UV light [100, 101]. In the presence of TiO_2_ at 300 nm irradiation, photocatalytic degradation of dicamba herbicide could be increased by 3 times compared with those without the addition of a TiO_2_ catalyst.  Figure 12 shows the degradation of methylene orange (MO) by PVP-assisted synthesized anatase TiO_2_ NPs.

**Figure 13:**
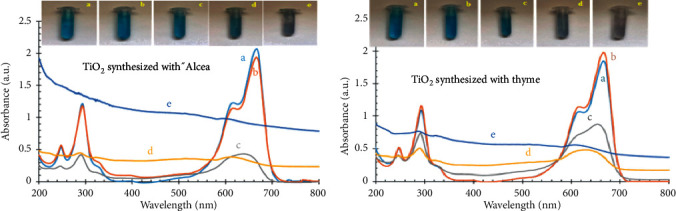
(a) Alcea- and (b) thyme-mediated biosynthesized TiO_2_ NPs for MB degradation [71].

The degradation of MO using a biogenic-synthesized TiO_2_ photocatalyst was investigated as shown in Figure 11. It is seen that a maximum of 94% degradation of MO was obtained at 1.0 g/dm^3^ under UV light within 150 min time allocated. The obtained results were attributed to an increase in the number of active sites and photons absorbed by the catalyst. Basically, as stated by Poudyal et al. [102], titanium dioxide nanoparticles are used as a catalyst with either fixed-phase or solvent-phase methods. Both techniques have their own advantages; however, the solvent phase is more effective than the fixed phase. Since the titanium dioxide NPs remain in the wastewater treatment process, it should be separated from the solution.  Figure 13 displays the UV-assisted application of titanium dioxide nanoparticles synthesized via the biogenic method using the extract of thyme and *Alcea* to remove methylene blue.

Figure 13 displays the degradation of MB before mixing (a), mixed for 30 min in a dark room (b), mixed for 30 min under UV irradiation (c), mixed for 60 min under UV irradiation (d), and mixed for 90 min under UV irradiation (*e*). The absorbance of the two synthesized TiO_2_ NPs under UV irradiation at different time intervals is depicted. As UV irradiation time increases, the absorption rate decreases. In fact, methylene blue was completely removed from the solution in both of the samples. There was no considerable change in the first 30 min of time interval; however, after 90 min irradiation, color change was observed. It was found that, by increasing the concentration of the catalyst to a specific amount, the efficiency improves. However, the excess concentration of the catalyst will have a negative effect on the radicals. This is because the catalyst particles prevent the penetration of the photons [103]. In addition to this, comparisons of the UV-Vis absorbance graphs of the two samples have indicated that TiO_2_ NPs synthesized with *thyme* could be chosen as a better photocatalyst than *alcea*-based ones. Therefore, the UV-visible analysis of photocatalytic properties confirmed the priority of TiO_2_ nanoparticles prepared with *thyme* extracts.

In addition to the removal of organic pollutants, TiO_2_ photocatalyst is also applicable for the removal of different inorganic pollutants present within the wastewater. TiO_2_ nanoparticles could be used to adsorb phosphates under UV irradiation; as supported by the work of Xie at al.[104], 95% of total phosphate was removed from surface water within 10 min. Similarly, removal of ammonia by a floating TiO_2_ system (immobilizing TiO_2_ nanoparticles with expanded clay aggregate granules) under solar light differences was noted on the fact that the effects of pH varied in ammonia removal [105].

## 5. Conclusions

Synthesis of nanoparticles involves the tailoring of materials at the atomic level to attain unique properties and manipulating for the desired applications. Synthesis of titanium nanoparticles would benefit from the development of clean, nontoxic, and environmentally acceptable green-chemistry procedures, probably involving organisms ranging from bacteria to fungi and algae to green plants. Green synthesis of TiO_2_ is a better method as they give excellent manipulation on controlling the particle size growth, thus provideing considerable stabilization during the TiO_2_ NPs synthesizing process. Currently, wastewater treatment using biogenic-assisted synthesized titanium nanometal oxides has gained great attention due to its high degradation efficiency, potential oxidation strength, high photostability, nontoxicity and noncorrosiveness, environmentally friendliness, long thermodynamic stability, abundancy, cost effectiveness, and its green nature.

## 6. Future Perspectives

It is obvious that titanium oxide nanoparticles could be synthesized using biogenic methods involving various biological sources. Synthesis of TiO_2_ NPs at large level/industrial level using green plants will lead to a hostile environment, and it involves the use of green parts of plants. Instead, in the future, researchers should adapt and focus on synthesis of TiO_2_ NPs using microorganisms such as fungi, algae, and bacteria by isolating from fertile and spoiled soil, characterization, culturing and use it as a template. To verify and enhance the degradation efficiency of organic dyes/pesticides and/or any water contaminants, researchers could focus on synthesizing of TiO_2_ NPs by considering different parameters that could affect the synthesis process and, simultaneously, degradation efficiency such as calcination temperature, pH, solvent effect, volume ratio between templates and the precursor salt, and concentration of the precursor.

## Figures and Tables

**Table 1:** Summary of the biogenic synthesis of TiO_2_ NPs using different templates.

Templates used	Size and morphology	Applications	References
*Kniphofia foliosa*	8.2–10.2, spherical	Antibacterial	[49]
*Calotropis gigantea*	10.52, spherical	Acaricidal	[56]
*Psidium guajava*	32.58	Antibacterial and antioxidant	[51]
*Aloe Barbadensis Miller*	20	—	[57]
*Ageratina alttissima L.*	60–100, spherical	Photocatalytic	[58]
*Vitex negundo* Linn.	10	Antibacterial	[59]
*Curcuma longa*	43.88, spherical	Biological property	[60]
*Vigna unguiculata*	11.55, spherical	Antimicrobial and cytotoxic effects	[61]
*Eclipta prostrata*	36, spherical	Antibacterial	[62]
*Nyctanthes arbor-tristis*	100–150, spherical and cubic	—	[49]
*Jatropha curcas L.*	25–100, spherical	—	[63]
*Albizia saman*	41, anatase crystal	—	[64]
*Cassia fistula*	38–44.2, spherical	—	[65]
*Taraxacum officinale* (yeast)	50, anatase and rutile	Photocatalytic	[66]
*Aeromonas hydrophila* (bacteria)	25–54	—	[43]
*Planomicrobium Bacillus mycoides* (bacteria)	8.89	—	[45]
*Moringa Oleifera*	40–60, spherical	—	[67]
*Aloe vera*	12 by XRD and 30 by PSA, tetragonal	—	[68]
Hibiscus flower	24.89 by XRD and 43.3 by PSA	—	[69]
Citrus lemon	2000 by SEM, different shapes	Antibacterial	[70]
*Alcea* and thyme	6 (thyme), 10 (alcea), polyhedron and irregular shape	Photocatalytic	[71]
Lignocellulosic waste	10–20 by XRD, 24.0 ± 4.7 nm and 13.0 ± 3.3 by TEM, anatase	—	[72]
*Echinacea purpurea herba*	120 by SEM	—	[73]
*Trigonella foenum-graecum*	25 by XRD and 20–90 by SEM, spherical	Antimicrobial	[74]
*Azadirachta indica*	56.13, spherical	—	[75]
*Bacillus mycoides* (bacterium)	40–60 nm, spherical	Green solar cell and antibacterial	[46]
*Bacillus subtilis* (bacterium)	10–30, spherical	Suppress aquatic biofilm growth	[76]
*Catharanthus roseus (Vinca rosea)*	25–110, irregular	Effective against hippobosca maculate and *Bovicola ovis*	[77]
*Annona squamosal*	40–60, spherical	—	[78]
*Planomicrobium sp.*	8.89 nm irregular, spherical	—	[79]
*Ocimum basilicum. L Var purapurascens benth-Lamicea*	6.97 by XRD and 50 by SEM, hexagon	—	[54]
Pristine pomegranate peel	92.8, anatase phase	Antimicrobial activity for water disinfection	[80]
*Garcinia zeylanica* extract	10, anatase and rutile phase	Antibacterial	[81]
*Cynodon dactylon*	13–34, irregular and hexagonal shape	Antibacterial and anticancer (A549 cell lines)	[82]

## Data Availability

All the necessary data are included in the review manuscript.

## References

[1] Sharma T. K., Yadav V. K., Shrivastav M., Dassani S., Singh R., Panday M. M. (2017). Biogenic synthesis, characterization and antibacterial effect of TiO_2_ nanoparticles. *International Journal of Current Research*.

[2] Arya S., Sonawan H., Math S. (2021). Biogenic titanium nanoparticles (TiO_2_ NPs) from *Tricoderma citrinoviride* extract: synthesis, characterization and antibacterial activity against extremely drug-resistant *Pseudomonas aeruginosa*. *International Nano Letters*.

[3] Hunagund S. M., Desai V. R., Kadadevarmath J. S., Barretto D. A., Vootla S., Sidarai A. H. (2016). Biogenic and chemogenic synthesis of TiO2NPs via hydrothermal route and their antibacterial activities, RSC Advances. *Royal Society of Chemistry Advances*.

[4] Fathima N. N., Aravindhan R., Rao J. R., Bu U. (2008). Dye house wastewater treatment through advanced oxidation process using Cu-exchanged Y zeolite: a heterogeneous catalytic approach. *Chemosphere*.

[5] Nair J. A., Hammes J., Cornelis G., Hassellöv M. (2016). Coagulation and sedimentation of gold nanoparticles and illite in model natural waters: influence of initial particle concentration. *NanoImpact*.

[6] Leudjo Taka A., Pillay K., Yangkou Mbianda X. (2017). Nanosponge cyclodextrin polyurethanes and their modification with nanomaterials for the removal of pollutants from waste water: a review. *Carbohydrate Polymers*.

[7] Dickhout J. M., Moreno J., Biesheuvel P. M., Boels L., Lammertink R. G. H., de Vos W. M. (2017). Produced water treatment by membranes: a review from a colloidal perspective. *Journal of Colloid and Interface Science*.

[8] Chatterjee D., Shimanti D. (2005). Visible light induced photocatalytic degradation of organic pollutants. *Journal of Photochemistry and Photobiology C: Photochemistry Review*.

[9] Gómez-Pastora J., Dominguez S., Bringas E., Rivero M. J., Ortiz I., Dionysiou D. D. (2017). Review and perspectives on the use of magnetic nanophotocatalysts (MNPCs) in water treatment. *Chemical Engineering Journal*.

[10] Chen F., Ho P., Ran R. (2017). Synergistic effect of CeO 2 modified TiO 2 photocatalyst on the enhancement of visible light photocatalytic performance. *Journal of Alloys and Compounds*.

[11] Kong H., Song J., Jang J. (2010). Photocatalytic antibacterial capabilities of TiO2−Biocidal polymer nanocomposites synthesized by a surface-initiated photopolymerization. *Environmental Science & Technology*.

[12] Deorsola F. A., Vallauri D. (2009). Study of the process parameters in the synthesis of TiO2 nanospheres through reactive microemulsion precipitation. *Powder Technology*.

[13] Carp O., Huisman C. L., Reller A. (2004). Photoinduced reactivity of titanium dioxide. *Progress in Solid State Chemistry*.

[14] Byrappa K., Yoshimura M. (2001). *Handbook of Hydrothermal Technology*.

[15] Mehdi E. M., Hamdi M., Meor Y. M. S., Wilfred P. (2013). Characterization of titania nanoparticles synthesized by the hydrothermal method with low grade mineral precursors. *Journal of Nanoparticle Research*.

[16] Kim C.-S., Moon B. K., Park J.-H., Choi B.-C., Seo H.-J. (2003). Solvothermal synthesis of nanocrystalline TiO2 in toluene with surfactant. *Journal of Crystal Growth*.

[17] Valencia S., Vargas X., Rios L., Restrepo G., Marín J. M. (2013). Sol-gel and low-temperature solvothermal synthesis of photoactive nano-titanium dioxide. *Journal of Photochemistry and Photobiology A: Chemistry*.

[18] Ishihara H., Bock J. P., Sharma R. (2010). Electrochemical synthesis of titania nanostructural arrays and their surface modification for enhanced photoelectrochemical hydrogen production. *Chemical Physics Letters*.

[19] Ahluwalia V. K. (2012). *Green Chemistry: Environmentally Benign Reactions*.

[20] Prathna T. C., Chandrasekaran N., Raichur A. M., Mukherjee A. (2011). Biomimetic synthesis of silver nanoparticles by Citrus limon (lemon) aqueous extract and theoretical prediction of particle size. *Colloids and Surfaces B: Biointerfaces*.

[21] Salata O. V. (2004). Applications of nanoparticles in biology and medicine. *Journal of Nanobiotechnology*.

[22] Mohanpuria P., Rana N. K., Yadav S. K. (2008). Biosynthesis of nanoparticles: technological concepts and future applications. *Journal of Nanoparticle Research*.

[23] Ir B., Muralitharan S., Ge S. (2005). Nanoparticle characterization of traditional homeopathically-manufactured gelsemium sempervirens medicines and Placebo Controls. *Journal of Nanomedicine & Biotherapeutic Discovery*.

[24] Saxena A., Tripathi R. M., Zafar F., Singh P. (2012). Green synthesis of silver nanoparticles using aqueous solution of *Ficus benghalensis* leaf extract and characterization of their antibacterial activity. *Materials Letters*.

[25] Taleb A., Petit C., Pileni M. P. (1997). Synthesis of highly monodisperse silver nanoparticles from AOT reverse micelles: a way to 2D and 3D self-organization. *Chemistry of Materials*.

[26] Mukherjee P., Ahmad A., Mandal D. (2001). Fungus-mediated synthesis of silver nanoparticles and their immobilization in the mycelial matrix: a novel biological approach to nanoparticle synthesis. *Nano Letters*.

[27] Rajakumar G., Rahuman A. A., Jayaseelan C. (2014). Solanum trilobatum extract-mediated synthesis of titanium dioxide nanoparticles to control Pediculus humanus capitis, Hyalomma anatolicum anatolicum and Anopheles subpictus. *Parasitology Research*.

[28] Ibrahem K. H., Salma J., Ali F. A. (2014). Effect of titanium nanoparticles biosynthesis by *lactobacillus on urease*, hemolysin & biofilm forming. *European Scientific Journal*.

[29] Marimuthu S., Rahuman A. A., Jayaseelan C. (2013). Acaricidal activity of synthesized titanium dioxide nanoparticles using calotropis gigantea against rhipicephalus microplus and haemaphysalis bispinosa. *Asian Pacific Journal of Tropical Medicine*.

[30] Madadi Z., Lotfabad T. B. (2016). Aqueous extract of acanthophyllum laxiusculum roots as a renewable resource for green synthesis of nano-sized titanium dioxide using sol-gel method. *Advanced Ceramics Progress*.

[31] Kashale A. A., Gattu K. P., Ghule K. (2016). Biomediated green synthesis of TiO2 nanoparticles for lithium ion battery application. *Composites Part B: Engineering*.

[32] Naik G. K., Mishra P. M., Parida K. (2013). Green synthesis of Au/TiO2 for effective dye degradation in aqueous system. *Chemical Engineering Journal*.

[33] Md Ishak N. A. I., Kamarudin S. K., Timmiati S. N. (2019). Green synthesis of metal and metal oxide nanoparticles via plant extracts: an overview. *Materials Research Express*.

[34] Khandel P., Yadaw R. K., Soni D. K., Kanwar L., Shahi S. K. (2018). Biogenesis of metal nanoparticles and their pharmacological applications: present status and application prospects. *Journal of Nanostructure in Chemistry*.

[35] Nadeem M., Tungmunnithum D., Hano C. (2018). The current trends in the green syntheses of titanium oxide nanoparticles and their Applications. *Green Chemistry Letters and Reviews*.

[36] Taoda H. (2008). Development of TiO2 photocatalysts suitable for practical use and their applications in environmental cleanup. *Research on Chemical Intermediates*.

[37] Narayanan K. B., Sakthivel N. (2011). Green synthesis of biogenic metal nanoparticles by terrestrial and aquatic phototrophic and heterotrophic eukaryotes and biocompatible agents. *Advances in Colloid and Interface Science*.

[38] Jha A. K., Prasad K., Kulkarni A. R. (2009). Synthesis of TiO2 nanoparticles using microorganisms. *Colloids and Surfaces B: Biointerfaces*.

[39] Abd El-Aziz A. R. M., AL-Othman M. R. (2013). Green synthesis of silver nanoparticles using *Aspergillus Terreus* (Kc462061). *Digest Journal of Nanomaterials and Biostructures*.

[40] Tarafdar A., Raliya R., Wang W.-N., Biswas P., Tarafdar J. C. (2013). Green synthesis of TiO2 nanoparticle using Aspergillus tubingensis. *Advanced Science, Engineering and Medicine*.

[41] Durairaj B., Xavier T., Muthu S. (2014). Fungal generated titanium dioxide nanoparticles: a potent mosquito (aedes aegypti) larvicidal agent. *Scholars Academic Journal of Biosciences*.

[42] Chokriwal A., Sharma M. M., Singh A. (2014). Biological synthesis of nanoparticles using bacteria and their applications. *American Journal of PharmTech Research*.

[43] Jayaseelan C., Rahuman A. A., Roopan S. M. (2013). Biological approach to synthesize TiO2 nanoparticles using Aeromonas hydrophila and its antibacterial activity. *Spectrochimica Acta Part A: Molecular and Biomolecular Spectroscopy*.

[44] Gupta S., Tripathi M. (2011). A review on the synthesis of TiO_2_ nanoparticles by solution route. *European Central Journal of Chemistry*.

[45] Malarkodi C., Chitra K., Rajeshkumar S. (2013). Novel eco-friendly synthesis of titanium oxide nanoparticles by using *planomicrobium sp*. and its antimicrobial evaluation. *Pelagia Research Library*.

[46] Aenishanslins N. A. O., Saona L. A., Durán-Toro V. M., Monrás J. P., Bravo D. M., Pérez-Donoso J. M. (2014). Use of titanium dioxide nanoparticles biosynthesized by bacillus mycoides in quantum dot sensitized solar cells. *Microbial Cell Factories*.

[47] Gopi D., Bhuvaneshwari N., Indira J., Kanimozhi K., Kavitha L. (2013). A novel green template assisted synthesis of hydroxyapatite nanorods and their spectral characterization. *Spectrochimica Acta Part A: Molecular and Biomolecular Spectroscopy*.

[48] Sundrarajan M., Gowri S. (2011). Green synthesis of titanium dioxide nanoparticles by nyctanthes arbor-tristis leaves extract. *Chalcogenide Letters*.

[49] Bekele E. T., Gonfa B. A., Zelekew O. A., Belay H. H., Sabir F. K. (2020). Synthesis of titanium oxide nanoparticles using root extract of *Kniphofia foliosa* as a template, characterization, and its application on drug resistance bacteria. *Journal of Nanomaterials*.

[50] Rajakumar G., Rahuman A. A., Roopan S. M. (2012). Fungus-mediated biosynthesis and characterization of TiO2 nanoparticles and their activity against pathogenic bacteria. *Spectrochimica Acta Part A: Molecular and Biomolecular Spectroscopy*.

[51] Santhoshkumar T., Rahuman A. A., Jayaseelan C. (2014). Green synthesis of titanium dioxide nanoparticles using *psidium guajava* extract and its antibacterial and antioxidant properties. *Asian Pacific Journal of Tropical Medicine*.

[52] Valli G., Geetha S. (2015). A green method for the synthesis of titanium dioxide nanoparticles using *cassia auriculata* leaves extract. *European Journal of Pharmaceutical Sciences*.

[53] Rao K. V., Kandregula G., Chinthakuntla A., Chidurala S., Rajendar V. (2015). Synthesis of TiO2 _n_anoparticles from o*range* fruit waste. *International Journal of Multidisciplinary Advanced Research Trends*.

[54] Salam H. A., Sivaraj R. (2014). Ocimum basilicum L. var. purpurascens Benth. *LAMIACEAE* mediated green synthesis and characterization of titanium dioxide nanoparticles. *Advances in Bioresearch*.

[55] Baker S., Rakshith D., Kavitha K. S. (2013). Plants: emerging as nanoparticles towards facile route in synthesis of nanoparticles. *BioImpacts*.

[56] Patil S. S. (2020). *Calotropis gigantea* assisted green synthesis of nanomaterials and their applications: a Review. *Journal of Basic and Applied Sciences*.

[57] Rao K. G., Ashok C. H., Rao K. V., Chakra C. S., Tambur P. (2015). Green synthesis of TiO_2_ nanoparticles using *Aloe vera* extract. *International Journal of Advanced Research in Physical Science (IJARPS)*.

[58] Ganesan S., Babu I. G., Mahendran D. (2016). Green engineering of titanium dioxide nanoparticles using Ageratina altissima (L.) King & H.E. Robines. medicinal plant aqueous leaf extracts for enhanced photocatalytic activity. *Annals of Phytomedicine: An International Journal*.

[59] Ambika S., Sundrarajan M. (2016). [EMIM] BF 4 ionic liquid-mediated synthesis of TiO_2_ nanoparticles using *Vitex negundo Linn* extract and its antibacterial activity. *Journal of Molecular Liquids*.

[60] Jalill R. D. H. A., Nuaman R. S., Abd A. N. (2016). Biological synthesis of titanium dioxide nanoparticles by *curcuma longa* plant extract and study its biological properties. *World Scientific News*.

[61] Chatterjee A., Ajantha M., Talekar A., Revathy N., Abraham J. (2017). Biosynthesis, antimicrobial and cytotoxic effects of titanium dioxide nanoparticles using *Vigna unguiculata* seeds. *International Journal of Pharmacognosy and Phytochemical Research*.

[62] Rajakumar G., Rahuman A. A., Priyamvada B., Khanna V. G., Kumar D. K., Sujin P. J. (2012). Eclipta prostrata leaf aqueous extract mediated synthesis of titanium dioxide nanoparticles. *Materials Letters*.

[63] Hudlikar M., Joglekar S., Dhaygude M., Kodam K. (2012). Green synthesis of TiO2 nanoparticles by using aqueous extract of Jatropha curcas L. latex. *Materials Letters*.

[64] Subhashini D., Valli Nachiyar C. (2014). Albizia saman: a Green route for the reduction of bulk TiO_2_. *International Journal of ChemTech Research*.

[65] Swathi N., Sandhiya D., Rajeshkumar S., Lakshmi T. (2019). Green synthesis of titanium dioxide nanoparticles using *cassia fistula* and its antibacterial activity. *International Journal of Research in Pharmaceutical Sciences*.

[66] Bao S.-J., Lei C., Xu M.-W., Cai C.-J., Jia D.-Z. (2012). Environment-friendly biomimetic synthesis of TiO_2_ nanomaterials for photocatalytic application. *Nanotechnology*.

[67] Patidar V., Jain P. (2017). Green synthesis of TiO_2_ nanoparticle using *Moringa oleifera* leaf extract. *International Research Journal of Engineering and Technology (IRJET)*.

[68] Khadar A., Behara D. K., Kumar M. K. (2016). Synthesis and characterization of controlled size TiO_2_ nanoparticles via green route using *Aloe vera* extract. *International Journal of Science and Research (IJSR)*.

[69] Rao K. G., Ashok C. H., Rao K. V., Chakra C. S., Rajendar V. Green synthesis of tio2 nanoparticles using hibiscus flower extract.

[70] Farook M. A., Mohamed H. S. M., Thomas J., Sathiyaseelan K., Venkatesan R., Kaviyarasu R. (2017). *Citrus lemon* leaf extract mediated synthesis of titanium dioxide nanoparticles. *Life Science Archives*.

[71] Arabi N., Kianvash A., Hajalilou A., Abouzari-Lotf E., Abbasi-Chianeh V. (2020). A facile and green synthetic approach toward fabrication of Alcea- and Thyme-stabilized TiO2 nanoparticles for photocatalytic applications. *Arabian Journal of Chemistry*.

[72] Ramimoghadam D., Bagheri S., Abd Hamid S. B. (2014). Biotemplated synthesis of anatase titanium dioxide nanoparticles via lignocellulosic waste material. *BioMed Research International*.

[73] Dobrucka R. (2017). Synthesis of titanium dioxide nanoparticles using *echinacea purpurea herba*. *Iranian Journal of Pharmaceutical Research*.

[74] Subhapriya S., Gomathipriya P. (2018). Green synthesis of titanium dioxide (TiO2) nanoparticles by Trigonella foenum-graecum extract and its antimicrobial properties. *Microbial Pathogenesis*.

[75] Kulkarni V., Palled V., Hiregoudar S., Prakash K. V., Maski D., lendra S. (2019). Bio-synthesis and characterization of titanium dioxide nanoparticles (TiO2) using Azadirachta indica leaf (neem leaf) extract. *International Journal of Current Microbiology and Applied Sciences*.

[76] Dhandapani P., Maruthamuthu S., Rajagopal G. (2012). Bio-mediated synthesis of TiO2 nanoparticles and its photocatalytic effect on aquatic biofilm. *Journal of Photochemistry and Photobiology B: Biology*.

[77] Velayutham K., Rahuman A. A., Rajakumar G. (2012). Evaluation of Catharanthus roseus leaf extract-mediated biosynthesis of titanium dioxide nanoparticles against Hippobosca maculata and Bovicola ovis. *Parasitology Research*.

[78] Thamima M., Karuppuchamy S. (2015). Biosynthesis of titanium dioxide and zinc oxide nanoparticles from natural sources: a review. *Advanced Science, Engineering and Medicine*.

[79] Ibrahem K. H., Salman J. A. S., Ali F. A. (2014). Effect of titanium nanoparticles biosynthesis by *lactobacillus crispatus* on urease, hemolysin& biofilm forming by some bacteria causing recurrent uti in iraqi women. *European Scientific Journal*.

[80] Abu-Dalo M., Jaradat A., Albiss B. A., Al-Rawashdeh N. A. F. (2019). Green synthesis of TiO_2_ NPs/Pristine *pomegranate* peel extract nanocomposite and its antimicrobial activity for water disinfection. *Journal of Environmental Chemical Engineering*.

[81] Senarathna U. L. N. H., Fernando S. S. N., Gunasekara T. D. C. P. (2017). Enhanced antibacterial activity of TiO2 nanoparticle surface modified with garcinia zeylanica extract. *Chemistry Central Journal*.

[82] Hariharan D., Srinivasan K., Nehru N. C. (2017). Synthesis and characterization of TiO_2_ nanoparticles using *cynodon dactylon* leaf extract for antibacterial and anticancer (A549 Cell Lines) Activity. *Journal of Nanomedicine Research*.

[83] Nwanya A. C., Ugwuoke P., Ejikeme P. M., Oparaku E. O. (2012). *Jathropha curcas* and *citrus aurantium* leaves dye extract for use in dye sensitized solar cell with TiO_2_ Films. *International Journal of Electrochemical Science*.

[84] Aryal S., Bahadur K. CR., Bhattard N., Kim CK., Kim HY. (2006). Study of electrolyte induced aggregation of gold nanoparticles capped by amino acids. *Journal of Colloids and Interface Science*.

[85] Roopan S. M., Bharathi A., Prabhakarn A. (2012). Efficient phyto-synthesis and structural characterization of rutile TiO2 nanoparticles using Annona squamosa peel extract. *Spectrochimica Acta Part A: Molecular and Biomolecular Spectroscopy*.

[86] Vijayalakshmi R., Rajendran V. (2012). Synthesis and characterization of nano-TiO_2_ via different methods. *Archives of Applied Science Research*.

[87] Ashok C., Venkateswara Rao K. (2014). ZnO/TiO2 nanocomposite rods synthesized by microwave-assisted method for humidity sensor application. *Superlattices and Microstructures*.

[88] Hajalilou A., Hashim M., Nahavandi M., Ismail I. (2014). Mechanochemical carboaluminothermic reduction of rutile to produce TiC-Al_2_O_3_ nanocomposite. *Advanced Powder Technology*.

[89] Pookmanee P., Phanichphant S. (2009). Titanium dioxide powder prepared by a sol-gel method. *Journal of Ceramic Processing Research*.

[90] Prasad K., Jha A. K., Kulkarni A. R. (2007). Lactobacillus assisted synthesis of titanium nanoparticles. *Nanoscale Research Letters*.

[91] Coenen K., Gallucci F., Mezari B., Hensen E., van Sint Annaland M. (2018). An in-situ IR study on the adsorption of CO2 and H2O on hydrotalcites. *Journal of CO2 Utilization*.

[92] Yenesew A., Dagne E., Müller M., Steglich W. (1994). An anthrone, an anthraquinone and two oxanthrones from *Kniphofia foliosa*. *Phytochemistry*.

[93] Peiró A. M., Peral J., Domingo C., Domènech X., Ayllón J. A. (2001). Low-temperature deposition of TiO2Thin films with photocatalytic activity from colloidal anatase aqueous solutions. *Chemistry of Materials*.

[94] Reddy G. B., Madhusudhan A., Ramakrishna D., Ayodhya D., Venkatesham M., Veerabhadram G. (2015). Green chemistry approach for the synthesis of gold nanoparticles with *gum kondagogu*: characterization, catalytic and antibacterial activity. *Journal of Nanostructure in Chemistry*.

[95] Linsebigler A. L., Lu G., Yates J. T. (1995). Photocatalysis on TiO2 surfaces: principles, mechanisms, and selected results. *Chemical Reviews*.

[96] Blanco J., Fernandez-Ibanez P., Malato S. (2007). Solar photocatalytic detoxification and disinfection of water: recent overview. *Journal of Solar Energy Engineering*.

[97] Prevot A. B., Vincenti M., Bianciotto A., Pramauro E. (1999). Photocatalytic and photolytic transformation of chloramben in aqueous solutions. *Applied Catalysis B: Environmental*.

[98] Doong R.-A., Maithreepala R. A., Chang S.-M. (2000). Heterogeneous and homogeneous photocatalytic degradation of chlorophenolsin aqueous titanium dioxide and ferrous ion. *Water Science & Technology*.

[99] Chong M. N., Jin B., Chow C. W. K., Saint C. (2010). Recent developments in photocatalytic water treatment technology: a Review. *Water Research*.

[100] Gaya U. I., Abdullah A. H. (2008). Heterogeneous photocatalytic degradation of organic contaminants over titanium dioxide: a Review of fundamentals, progress and problems. *Journal of Photochemistry and Photobiology C: Photochemistry Reviews*.

[101] Fujishima A., Rao T. N., Tryk D. A. (2002). Titanium dioxide photocatalysis. *Journal of Photochemistry and Photobiology C: Photochemistry, Reviews*.

[102] Poudyal K., Clark D., Brag A. (2002). Titanium dioxide photocatalysis of metals. *Enve Bull.: Hazard. Waste Management*.

[103] Kuo W. S., Ho P. H. (2001). Solar photocatalytic decolorization of methylene blue in water. *Chemosphere*.

[104] Xie H., Zheng Q., Wang S. (2014). Capture of phosphates in surface water by TiO_2_ nanoparticles under UV irradiation. *Particuology*.

[105] Shavisi Y., Sharifnia S., Zendehzaban M., Mirghavami M. L., Kakehazar S. (2014). Application of solar light for degradation of ammonia in petrochemical wastewater by a floating TiO_2_/LECA photocatalyst. *Journal of Industrial and Engineering Chemistry*.

